# Machine learning predicts lung recruitment in acute respiratory distress syndrome using single lung CT scan

**DOI:** 10.1186/s13613-023-01154-5

**Published:** 2023-07-05

**Authors:** Francesca Pennati, Andrea Aliverti, Tommaso Pozzi, Simone Gattarello, Fabio Lombardo, Silvia Coppola, Davide Chiumello

**Affiliations:** 1 Ipartimento di Elettronica, Informazione e Bioingegneria, Politecnico di Milano, Milan, Italy; 2 Department of Health Sciences, University of Milan, Milan, Italy; 3 Department of Anesthesiology, University Medical Center Göttingen, Göttingen, Germany; 4 Department of Anesthesia and Intensive Care, ASST Santi Paolo e Carlo, San Paolo University Hospital, Via Di Rudini 9, Milan, Italy; 5 Coordinated Research Center on Respiratory Failure, University of Milan, Milan, Italy

**Keywords:** ARDS, Machine learning, Tomography

## Abstract

**Background:**

To develop and validate classifier models that could be used to identify patients with a high percentage of potentially recruitable lung from readily available clinical data and from single CT scan quantitative analysis at intensive care unit admission. 221 retrospectively enrolled mechanically ventilated, sedated and paralyzed patients with acute respiratory distress syndrome (ARDS) underwent a PEEP trial at 5 and 15 cmH_2_O of PEEP and two lung CT scans performed at 5 and 45 cmH_2_O of airway pressure. Lung recruitability was defined at first as percent change in not aerated tissue between 5 and 45 cmH_2_O (radiologically defined; recruiters: Δ_45-5_non-aerated tissue  > 15%) and secondly as change in PaO_2_ between 5 and 15 cmH_2_O (gas exchange-defined; recruiters: Δ_15-5_PaO2  > 24 mmHg). Four machine learning (ML) algorithms were evaluated as classifiers of radiologically defined and gas exchange-defined lung recruiters using different models including different variables, separately or combined, of lung mechanics, gas exchange and CT data.

**Results:**

ML algorithms based on CT scan data at 5 cmH_2_O classified radiologically defined lung recruiters with similar AUC as ML based on the combination of lung mechanics, gas exchange and CT data. ML algorithm based on CT scan data classified gas exchange-defined lung recruiters with the highest AUC.

**Conclusions:**

ML based on a single CT data at 5 cmH_2_O represented an easy-to-apply tool to classify ARDS patients in recruiters and non-recruiters according to both radiologically defined and gas exchange-defined lung recruitment within the first 48 h from the start of mechanical ventilation.

**Supplementary Information:**

The online version contains supplementary material available at 10.1186/s13613-023-01154-5.

## Key points

- Machine learning based on a single CT imaging at the admission in intensive care unit provided a reliable tool to classify ARDS patients in responder and not responder to lung recruitment within the first 48 hours from the start of mechanical ventilation.

**Supplementary Information:**

The online version contains supplementary material available at 10.1186/s13613-023-01154-5.

## Introduction

ARDS is typically defined as a non-cardiogenic pulmonary edema characterized by different degree of hypoxemia, alveolar shunt and not aerated lung regions [1]. The commonly suggested lung protective ventilation strategy includes lung recruitment maneuvers to reopen not-aerated lung regions (i.e., collapsed areas) in order to reduce VILI, improve lung oxygenation and CO_2_ removal [2]. However, several data showed that lung recruitment, although improving the oxygenation, could also, at the same time, impair the hemodynamics without improving the 28-day mortality [3, 4]. Furthermore, a systematic review and meta-analysis reported that in patients with moderate–severe ARDS, the use of higher PEEP with prolonged lung recruitment was associated with increased risk of death compared to similar PEEP without lung recruitment [5]. Thus, selecting an appropriate ventilatory strategy balancing the levels of PEEP and lung recruitment is therefore critical.

Among the different lung imaging techniques, the CT is the reference method both for a morphological analysis and for an accurate quantitative computation of lung recruitability [6–8]. The measurement of lung potential recruitment is fundamental to establish the therapeutic efficacy of PEEP [8–10]. It has been reported that the amount of lung recruitability ranged from 0 up to 70% of the total lung weight [8, 10]. Moreover, the lung recruitability was poorly predictable, being affected by the distribution of the lung disease, amount of edema, timing of ARDS onset and alteration in respiratory mechanics [9–12].

Recently, several applications of machine learning techniques have been applied in critical care medicine with promising results [13, 14]. Machine learning algorithms have been proposed to classify patients into ARDS subphenotypes using readily available clinical data [15–19]. Various studies demonstrated that machine learning can be used to predict patients who required prolonged mechanical ventilation and also the outcome [14, 15]. In a secondary analysis of a randomized trial applying machine learning, three different ARDS clusters were found, differing in the injury effect of an open lung recruitment strategy and the outcome [20]. Recently, several prediction models of COVID-19 have been also developed with a focus on CT diagnosis and prognosis [21–25].

Thus, we hypothesized to apply machine learning algorithms for the detection of lung recruitment, defined both from radiological and gas exchange data, in ARDS patients. In particular, the aim of the present study was to develop and validate classifier models to identify patients with a high percentage of potentially recruitable lung from readily available clinical data (namely mechanics and gas exchange) and using single CT scan at admission at 5 cmH_2_O of PEEP. The secondary aim was to develop models that use a more limited set of available clinical and CT scan data.

## Materials and methods

The study is a retrospective analysis of ARDS patients previously enrolled from 2016 to December 2022 and partially included in other already published studies [26]. The study was approved by the Institutional Review board of our hospital (Comitato Etico Interaziendale Milano Area A, protocol number 2016/ST/143 on the 22nd June 2016, entitled “PEEP test”) and informed consent was obtained according to the Italian regulations. The study protocol flowchart is shown in Additional file 1: Figure S1.

### Study protocol

At Intensive Care Unit (ICU) admission, patients were maintained deeply sedated and paralyzed, ventilated in volume control ventilation, with a tidal volume between 6 and 8 ml/kg of ideal body weight, a respiratory rate to ensure an arterial carbon dioxide partial pressure (PaCO_2_) between 40 and 50 mmHg; positive end-expiratory pressure (PEEP) and FiO_2_ were set by the attending physician to ensure an arterial saturation between 88 and 92%. An esophageal balloon catheter (Smart Cath, Viasys, Palm Springs, USA) was placed in the lower third of the esophagus, as already described before [27].

Patients were enrolled within 48 h from ICU admission and the study protocol was started.

At the beginning of the study, a recruitment maneuver was performed in pressure controlled ventilation at PEEP 5 cmH_2_O, with a plateau pressure of 45 cmH_2_O, I:E 1:1, respiratory rate of 10 breaths/min for 2 min. Subsequently, the previously applied tidal volume and respiratory rate were resumed and a PEEP trial at 5 and 15 cmH_2_O was performed; the FiO_2_ was adjusted at the beginning of the trial to ensure an arterial saturation between 88 and 92%. At each PEEP level, after 20 min, end-inspiratory and end-expiratory pauses were performed and arterial and central venous blood gas analysis were obtained; consequently, we recorded partitioned respiratory mechanics, gas exchange and hemodynamics variables. Partitioned respiratory mechanics of lung and chest wall elastance was computed according to the following standard formulas [9]. Gas exchange-defined lung recruitment was assessed as the difference in PaO_2_ between 15 and 5 cmH_2_O of PEEP.

After the PEEP trial, two whole lung CT scan in static condition at 5 cmH_2_O of end-expiratory airway pressure and 45 cmH_2_O of end-inspiratory airway pressure were performed.

#### Lung CT quantitative analysis

An integrated approach was used based on manual segmentation of the lung by a dedicated software and subsequently automatically analyzed (Soft-E-Film). The total lung weight, the gas volume, and the amount of the different compartment (not inflated, poor inflated, well inflated and overinflated) were computed [6]. Radiologically defined lung recruitment was assessed as the ratio between the difference in not aerated tissue at 5 cmH_2_O and 45 cmH_2_O of airway pressure to the total lung tissue weight at 5 cmH_2_O of airway pressure.

### Statistical analysis

Continuous variables are presented as mean ± standard deviation or median (interquartile range), as appropriate, whereas categorical data are reported as percentages. Clinical data of recruiters and non-recruiters at 5 cmH_2_O, as well as the differences in respiratory mechanics and gas exchange between 5 cmH_2_O and 45 cmH_2_O of airway pressures were compared by the Student’s t test or Mann–Whitney rank-sum test, as appropriate. Categorical data were compared by the Chi-square test. Tests were two-sided with significance α level set at less than 0.05.

#### Machine learning models

**Fig. 1:**
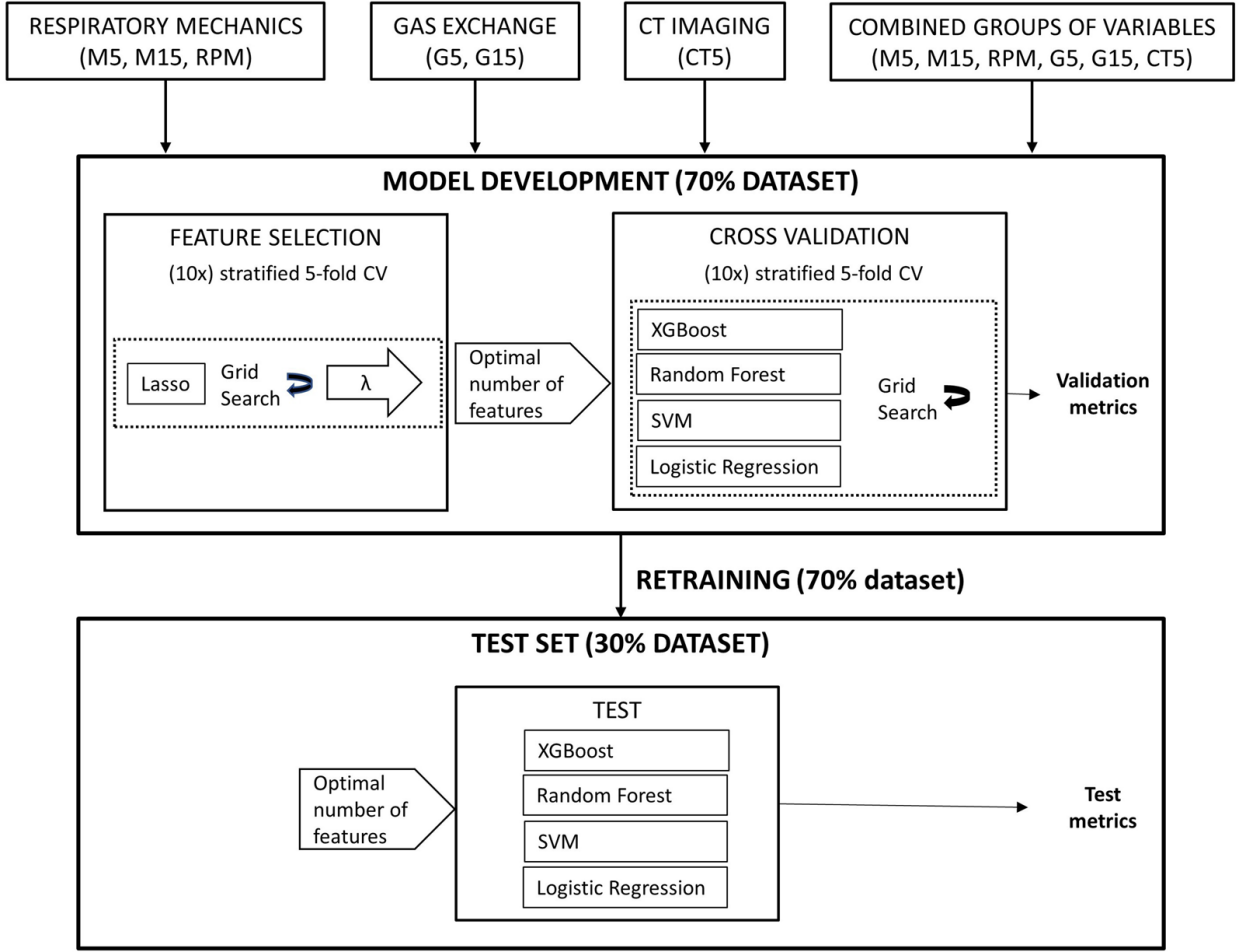
The machine learning workflow. Input parameters included lung mechanics at PEEP 5 cmH_2_O (M5), lung mechanics at PEEP 15 cmH_2_O (M15), respiratory partitioned mechanics (RPM), gas exchange measured at PEEP 5 cmH_2_O (G5), gas exchange measured at PEEP 15 cmH_2_O (G15), CT imaging acquired at PEEP 5 cmH_2_O (CT5). A grid search strategy with a stratified fivefold cross-validation repeated 10 times was performed to optimize algorithms’ parameters, for both feature selection and model training. The hold-out test set was used to test the re-trained models in terms of mean area under the receiver operating characteristic curve (AUC), accuracy, sensitivity and specificity

Machine learning models were implemented in Python using the Scikit-Learn package [28]. Data management was performed using the Pandas library [29]. The workflow is summarized in Fig. 1.

##### Outcome

The outcome of interest was the prediction of patients with a percentage of potentially recruitable lung greater than the median value of the whole population. Radiologically defined lung recruitability was assessed as the ratio of the change in not aerated lung tissue between 5 cmH_2_O and 45 cmH_2_O to the total lung tissue weight at 5 cmH_2_O at CT scan. Gas exchange-defined lung recruitment was assessed as the difference in PaO_2_ between 15 and 5 cmH_2_O of PEEP (Δ_15-5_ PaO_2_)_._ For the two outcomes, the median values for the whole population were 15% and 24 mmHg, respectively. Furthermore, to evaluate the model performance over different lung recruitability thresholds, the analyses on the overall dataset was repeated for all models by assigning classes using cut-offs of 10%, 20% and 30% for radiologically defined lung recruitability and using cut-offs of 20 mmHg, 30 mmHg and 40 mmHg for gas exchange-defined lung recruitability.

##### Predictor variables

With the rationale of investigating the best measuring conditions in the clinical setting, classifier models were developed using demographic data with the addiction of sparse sets of variables that were grouped according to the variable type: lung mechanics (M), gas exchange (G) and CT imaging data acquired at PEEP 5 cmH_2_O (CT5). Lung mechanics and gas exchange variable sets were further subdivided according to the measuring condition: lung mechanics at PEEP 5 cmH_2_O (M5), lung mechanics at PEEP 15 cmH_2_O (M15), respiratory partitioned mechanics (RPM), gas exchange measured at PEEP 5 cmH_2_O (G5) and gas exchange measured at PEEP 15 cmH_2_O (G15). A total of 44 features were used as independent variables for the development of the models (Additional file 1: Figure S2).

##### Data pre-processing

To ensure the availability of all predictors in models’ development, we excluded features with more than 30% missing data. In the remaining features, residual missing data were imputed with the median value of the respective feature. Finally, the data were normalized and scaled to have zero mean and unit variance such that variables with different scales can contribute equally to the analysis. For the purposes of evaluation, we reserved 30% of the dataset, chosen at random, as a hold-out dataset and used the remaining 70% to train, validate, and iterate the predictive models. The hold-out dataset was used to assess the performance of the models on totally unseen data. As imbalanced class distribution can affect model performance, the Synthetic Minority Oversampling Technique (SMOTE) was applied during training to balance the dataset [30].

##### Feature selection

The purpose of this step is to find the smallest number of relevant and informative features. In the training set, the least absolute shrinkage and selection operator (LASSO) was repeatedly applied, each time with a different random data split, and the features that had been selected in more than 50% of the case were retained. Additional details on feature selection are reported in Additional file 1: Section S3.

##### Machine learning classification algorithms (classifiers)

Four conventional ML algorithms were implemented to classify recruiters, considering their robustness in binary prediction problems. Logistic regression (LR) is a widely used machine learning model in medicine for classification tasks, which assumes a linear relationship between the input variables and the outcomes. Support Vector Machine (SVM) is a maximum margin classifier that performs classification by finding a decision boundary, which generates the maximum separation between decision classes [31]. Random Forest [32] and XGBoost [33] are two ensemble techniques, respectively, a bagging and a boosting type of ensemble, characterized by high generalizability and robustness, which are effective at capturing interactions and non-linear relationships between variables, by aggregating sub-models that have no or low correlation with each other [34, 35]. During models’ development, a fivefold cross-validation (stratified fivefold CV) routine was defined so that the data were partitioned into five folds of equal size: training occurred on four of the folds, and the remaining fold was used as validation set, to monitor the performance of the algorithm. Folds were created 10 times, each time with a different data split, to remove any bias in selecting training and validation subsets (repeated (stratified) fivefold CV). Models’ hyper-parameters were defined through a cross-validated grid search, as the combination of parameters that maximized models’ performance: for each model, various combinations of parameters were tried and the one with the best cross-validation accuracy was selected. As recommended by Hsu et al. [36], a coarse grid was first used to identify the “best region” of each parameter, followed by a finer grid within this region. This parameter search has been done for each set of features, as the parameters’ setting may vary with different set of features.

The median value of the area under the receiver operating characteristic curve computed from the validation folds (validation AUC) was chosen as the summarizing metric. The comparison between the percentages of patients having high or low lung recruitability, based on the different classification models, was performed with the Cochran's Q test. When applicable, significant differences between the percentages were tested using the McNemar test. These tests were implemented using the python library mlxtend. The model with the best validation AUC was retained and its performance was evaluated on the test set, which was kept isolated from the model development process, by calculating AUC, accuracy, sensitivity and specificity.

## Results

A total of 221 patients were retrospectively analyzed [9]. According to the median value of radiologically defined and gas exchange-defined recruitability, recruiters and non-recruiters were 110 and 111, respectively. The main clinical characteristics of the population at 5 cmH_2_O of PEEP divided in recruiters (*n* = 110) and not recruiters (*n* = 111) according to radiologically defined lung recruitability are reported in Table 1. The recruiter group presented a significantly higher percentage of pulmonary ARDS origin compared to the non-recruiter (73% *vs* 51%). The recruiter group was ventilated with a significantly lower tidal volume compared to non-recruiters (500 [425–560] vs 522 [461–600] mL) but with a similar minute ventilation. At 5 cmH_2_O of PEEP, respiratory system and lung elastances were both significantly higher in recruiters (27 [6, 19–32] vs 24 [6, 17–27] cmH_2_O/L and 21 [12–26] vs 18 [11–22] cmH2O/L, respectively). Arterial oxygenation (PaO_2_/FiO_2_) was significantly lower in recruiters compared to non-recruiters (113 [84–144] vs 163 [119–207]). At 5 cmH_2_O of PEEP, recruiters had a lower lung gas volume and higher lung weight compared to non-recruiters (736 [504–979] vs 1233 [912–1798] mL and 1598 [1278–1968] vs 1319 [1116–1472] g). Similarly, the percentage of not aerated and well-aerated tissue was, respectively, higher and lower in recruiters compared to non-recruiters (52 ± 14 vs 35 ± 14% and 14 [8, 10–20] vs 34 [6, 11, 24–38] %).

**Table 1:** Baseline characteristics at 5 cmH_2_O of PEEP in patients divided according to lung potential recruitment (LPR)

Variables	Recruiters (LPR > 15%)	Non-recruiters (LPR ≤ 15%)	*p*-value
Demographic			
Number	110	111	
Age (years)	62 (47, 74)	61 (48, 72)	0.317
Male sex (%)	44	56	0.739
Weight (kg)	75 (61, 85)	75 (61, 89)	0.718
BMI (kg/m2)	25 (22, 28)	25 (22, 29)	0.894
SAPS II	43 ± 17	42 ± 13	0.406
Origin of ARDS			**0.001**
Pulmonary (%)	73	51	
Extrapulmonary (%)	27	49	
ARDS severity			** < 0.001**
Mild (%)	61	27	
Moderate (%)	28	42	
Severe (%)	11	31	
Ventilatory parameters			
Tidal volume (TV, ml)	500 (425, 560)	522 (461, 600)	**0.013**
TV per ideal body weight (mL/kg)	7.9 (6.7, 8.5)	8.0 (7.3, 9.2)	**0.017**
Respiratory rate (breath per minute)	16 (14, 20)	16 (14, 18)	0.541
Minute ventilation (L/min)	8.3 (6.9, 9.9)	8.4 (7.6, 10.0)	0.252
Peak inspiratory pressure (cmH_2_O)	28 (23, 34)	26 (23, 29)	**0.029**
Plateau pressure (cmH_2_O)	19 (16, 22)	18 (15, 20)	**0.030**
Physiological dead space	0.66 ± 0.13	0.57 ± 0.12	** < 0.001**
Respiratory mechanics			
Driving pressure (cmH_2_O)	13 (10, 16)	12 (10, 15)	0.112
Respiratory system elastance (cmH_2_O/L)	27 (21, 35)	24 (19, 30)	**0.007**
Mechanical power (J/(min kg))	15 (11, 20)	15 (11, 18)	0.890
Respiratory partitioned mechanics			
Lung elastance (cmH_2_O/L)	21(14, 28)	18 (13, 24)	**0.048**
Chest wall elastance (cmH_2_O/L)	6 (4, 9)	6 (4, 8)	0.995
Gas exchange			
PaCO_2_ (mmHg)	47 (41, 53)	42 (38, 49)	** < 0.001**
PaO_2_ (mmHg)	68 (60, 75)	76 (65, 91)	** < 0.001**
PaO_2_/FiO_2_	113 (84, 144)	163 (119, 207)	** < 0.001**
CT parameters			
Total lung volume (mL)	2377 (2073, 2798)	2701 (2056, 3201)	0.022
Total lung weight (g)	1598 (1278, 1968)	1319 (1116, 1472)	** < 0.001**
Total lung gas volume (mL)	736 (504, 979)	1233 (912,1798)	** < 0.001**
Not aerated lung tissue (%)	52 ± 14	35 ± 14	** < 0.001**
Poorly aerated lung tissue (%)	31 (21, 39)	28 (22, 39)	0.652
Well aerated lung tissue (%)	14 (10, 22)	34 (26, 42)	** < 0.001**
Overaerated lung tissue (%)	0.00 (0.00, 0.04)	0.03 (0.00, 0.29)	** < 0.001**

Continuous data are expressed as mean ± SD or median (interquartile range), while categorical data are expressed as %. Student’s t test or Mann–Whitney rank-sum tests and Chi-square test were used as appropriate

The PEEP test response of the population divided according to radiologically defined lung recruitability is shown in Additional file 1: Table S1 and described in Additional file 1: Section S2.

### Development of classification models

From set of predictor variables (M5, M15, RPM, G5, G15, CT5), using LASSO, subsets of the most informative variables, were produced and used in building ML models, and are summarized in Table 2. See also Additional file 1: Section S3.

**Table 2:** Subsets of the most informative variables selected according to the frequency with which they were chosen after repeating the least absolute shrinkage and selection operator (LASSO) algorithm

Feature group name	Data
Outcome: Δ_45-5_non-aerated tissue > 15%	
M5	ARDS origin, tidal volume, plateau pressure, driving pressure
M5 + M15	ARDS origin, tidal volume, plateau pressure, driving pressure, Δ_15-5_ mechanical power
M5 + M15 + RPM	ARDS origin, tidal volume, plateau pressure, driving pressure, Δ_15-5_ mechanical power, lung elastance
G5	ARDS origin, PaO_2_/FiO_2_
G5 + G15	ARDS origin, PaO_2_/FiO_2_, Δ_15-5_ PaO_2_
CT5	Age, ARDS origin, well-aerated lung tissue, non-aerated lung tissue
CT5 + G5	Age, ARDS origin, well-aerated lung tissue, non-aerated lung tissue, PaO_2_/FiO_2_
CT5 + M5	Age, ARDS origin, well-aerated lung tissue, non-aerated lung tissue
CT5 + G5 + G15	Age, ARDS origin, well-aerated lung tissue, non-aerated lung tissue, Δ_15-5_ PaO_2_
CT5 + M5 + M15 + RPM	Age, ARDS origin, well-aerated lung tissue, non-aerated lung tissue, Δ_15-5_ mechanical power
CT5 + G5 + G15 + M5 + M15 + RPM	Age, ARDS origin, well-aerated lung tissue, non-aerated lung tissue, Δ_15-5_ PaO_2_, Δ_15-5_ mechanical power
Outcome: Δ_15-5_PaO2 > 24 mmHg	
M5	ARDS origin, BMI, mechanical power
M5 + M15	ARDS origin, BMI, mechanical power, Δ_15-5_ driving pressure
M5 + M15 + RPM	ARDS origin, BMI, mechanical power, Δ_15-5_ driving pressure, chest wall elastance
G5	ARDS origin, BMI, PaO_2_
CT5	ARDS origin, total lung weight, poorly aerated lung tissue, well-aerated lung tissue
CT5 + G5	ARDS origin, total lung weight, poorly aerated lung tissue, well-aerated lung tissue, PaO_2_
CT5 + M5	ARDS origin, total lung weight, poorly aerated lung tissue, well-aerated lung tissue, mechanical power
CT5 + M5 + M15 + RPM	ARDS origin, total lung weight, poorly aerated lung tissue, well-aerated lung tissue, Δ_15-5_ driving pressure, chest wall elastance
CT5 + G5 + M5 + M15 + RPM	ARDS origin, total lung weight, poorly aerated lung tissue, well-aerated lung tissue, Δ_15-5_ driving pressure, chest wall elastance, PaO_2_
M5	ARDS origin, BMI, mechanical power
CT5 + G5 + G15 + M5 + M15 + RPM	Age, ARDS origin, well-aerated lung tissue, non-aerated lung tissue, Δ_15-5_ PaO_2_, Δ_15-5_ mechanical power

Lung recruitability was defined both as the percent change in not aerated tissue between 5 cmH_2_O and 45 cmH_2_O (recruiters: Δ_45-5_non-aerated tissue > 15%) and as the change in PaO_2_ between 5 cmH_2_O and 15 cmH_2_O (recruiters: Δ_15-5_PaO2 > 24 mmHg). Input parameters included lung mechanics at PEEP 5 cmH_2_O (M5), lung mechanics at PEEP 15 cmH_2_O (M15), respiratory partitioned mechanics (RPM), gas exchange measured at PEEP 5 cmH_2_O (G5), gas exchange measured at PEEP 15 cmH_2_O (G15), CT imaging acquired at PEEP 5 cmH_2_O (CT5)

### Model performances

#### Radiologically defined lung recruitability: ML algorithm selection

**Fig. 2:**
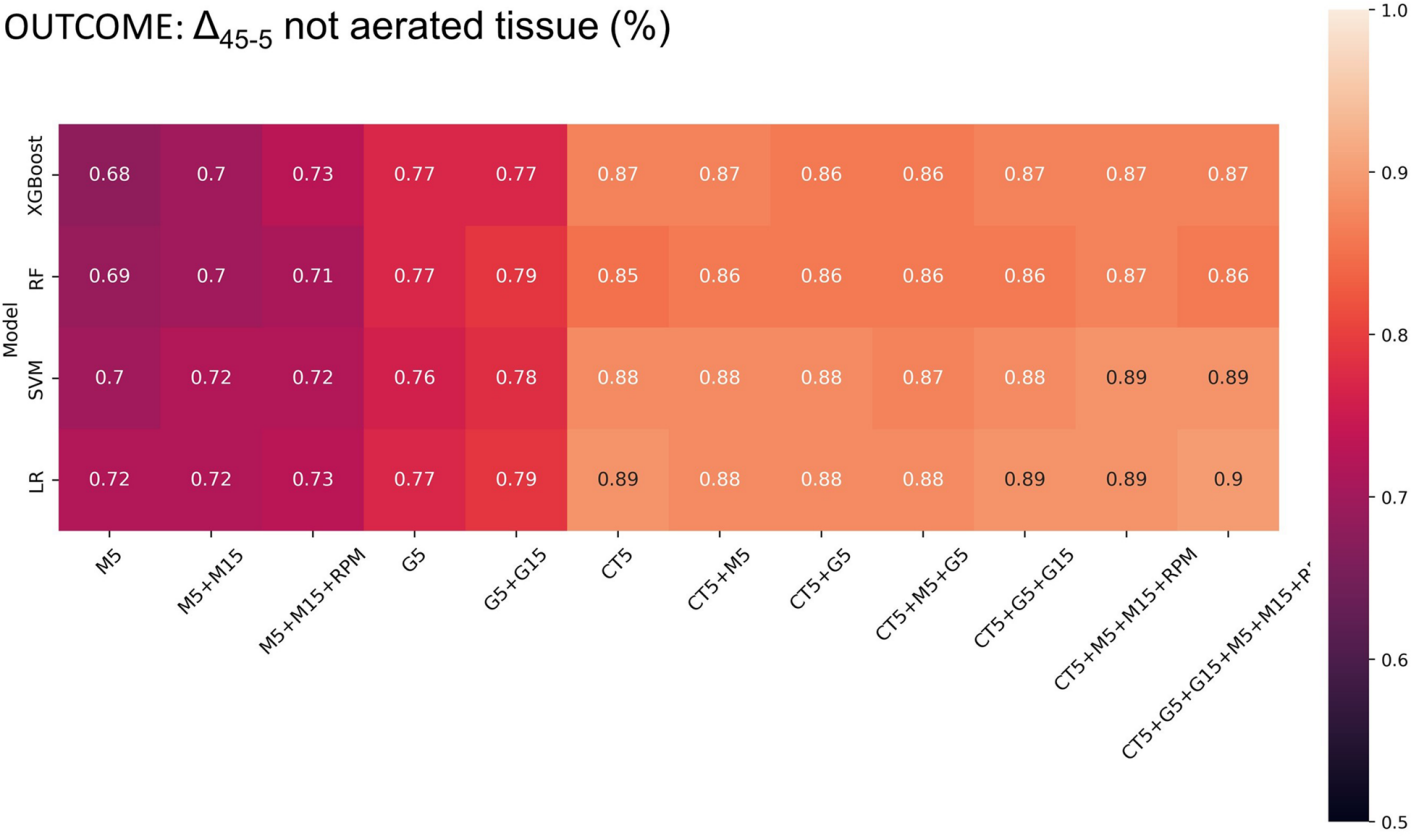
Validation AUC for each pair of dataset and machine learning algorithm, when lung recruitability was radiologically defined (recruiters: Δ_45-5_non-aerated tissue > 15%). M5, lung mechanics at PEEP 5 cmH_2_O, M15, lung mechanics at PEEP 15 cmH_2_O, RPM, respiratory partitioned mechanics, G5, gas exchange measured at PEEP 5 cmH_2_O, G15, gas exchange measured at PEEP 15 cmH_2_O, CT5, CT imaging acquired at PEEP 5 cmH_2_O. XGBoost, gradient-boosted tree; RF, random forest; LR, logistic regression; SVM, support vector machine

Figure 2 shows the validation AUCs for each pair of set of variables and ML algorithm, when lung recruitability was radiologically defined (recruiters: Δ_45-5_non-aerated tissue > 15%). More details on the comparison between the classifiers are reported in the online Additional file 1: Section S4. Based on the results, logistic regression (LR) was chosen as ML classification algorithm (classifier) when lung recruitability was radiologically defined, as faster and more interpretable compared to the other algorithms. Additional metrics for the logistic regression algorithm are reported in Additional file 1: Table S2.

### Radiologically defined lung recruitability: logistic regression performance on different set of variables

Figure 2 shows that models based only on lung mechanics and gas exchange variables showed worse AUCs, with the highest values achieved by the models trained on gas exchange variables measured at PEEP 5 cmH_2_O and 15 cmH_2_O (0.79 for the LR model). The Cochran test reported no statistically significant difference among mechanical models (*p* = 0.920), among gas exchange models (*p* = 0.317) and between mechanical and gas exchange models (*p* = 0.893). The model based on all features (CT5 + G5 + G15 + M5 + M15 + RPM) showed the highest validation AUC (0.90), but no significant difference was obtained when only CT data model (CT5; AUC 0.89) was evaluated (*p* = 0.466). Models including CT parameters reported statistically significant higher AUCs compared to models based only on lung mechanics (M5, M5 + M15, M5 + M15 + RPM) and gas exchange (G5, G5 + G15) (*p* < 0.001) (Fig. 2).

### Gas exchange-defined lung recruitability: ML algorithm selection

**Fig. 3:**
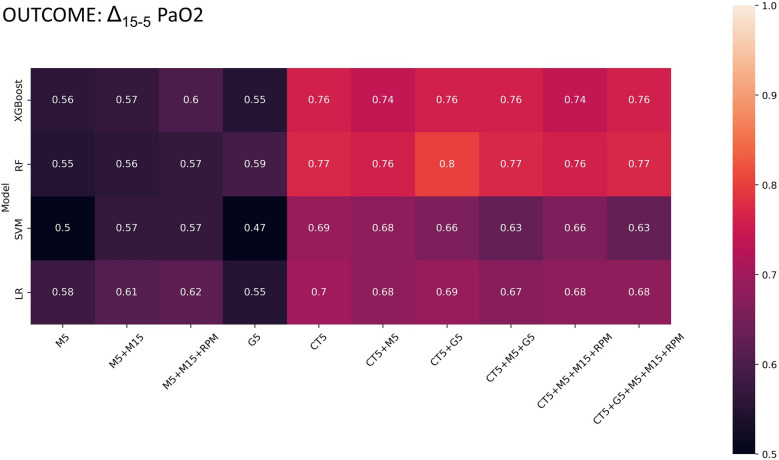
Validation AUC for each pair of dataset and machine learning algorithm, when lung recruitability was gas exchange-defined (recruiters: Δ_15-5_PaO_2_ > 24 mmHg). M5, lung mechanics at PEEP 5 cmH_2_O, M15, lung mechanics at PEEP 15 cmH_2_O, RPM, respiratory partitioned mechanics, G5, gas exchange measured at PEEP 5 cmH_2_O, CT5, CT imaging acquired at PEEP 5 cmH_2_O. XGBoost, gradient-boosted tree; RF, random forest; LR, logistic regression; SVM, support vector machine

When lung recruitability was defined based on gas exchange (recruiters: Δ_15-5_PaO_2_ > 24 mmHg) (Fig. 3), random forest resulted in the statistically highest validation AUCs on gas exchange and CT models and was chosen as classification algorithm. More details on the comparison between the classifiers is reported in the online Additional file 1: Section S4. Additional metrics for the random forest algorithm are reported in Additional file 1: Table S3.

### Gas exchange-defined lung recruitability: random forest performance on different set of variables

Figure 3 shows that random forest algorithm based only on lung mechanics (M5, M5 + M15, M5 + M15 + RPM) and gas exchange variables (G5) showed AUCs lower than 0.6. No statistically significant difference among mechanical models was found (*p* = 0.108). Significant difference was reported among models including CT data (*p* = 0.005), with better performance of CT5 (AUC 0.77) compared to CT5 + M5 (AUC 0.76) (McNemar’s test *p* = 0.032). Models based on CT parameters reported statistically significant higher AUCs compared to models based only on lung mechanics (*p* = 0.009) and gas exchange (*p* = 0.035).

The models’ performance over a range of thresholds of radiologically defined and gas exchange-defined lung recruitment is presented in Table 3 using the overall dataset (CT5 + G5 + G15 + M5 + M15 + RPM). For radiologically defined lung recruitment, similar performance was obtained from logistic regression when lung recruitability is defined using a cut-off 10% and 20%, whereas sensitivity worsened when 30% was used as threshold.

**Table 3:** Models’ performance over a range of thresholds defining lung recruitment

	Validation				Test			
	AUC	acc	Sens	Spec	AUC	acc	Sens	Spec
Outcome: Δ_45-5_non-aerated tissue								
> 10% (*n* = 142)	0.85(0.07)	0.77(0.07)	0.78(0.10)	0.76(0.10)	0.90	0.81	0.80	0.82
> 20% (*n* = 76)	0.88(0.05)	0.81(0.06)	0.81(0.12)	0.81(0.08)	0.84	0.78	0.75	0.79
> 30% (*n* = 32)	0.89(0.07)	0.82(0.07)	0.81(0.18)	0.82(0.08)	0.85	0.87	0.37	0.93
Outcome: Δ_15-5_PaO2								
> 20 mmHg (*n* = 124)	0.78(0.07)	0.71(0.07)	0.71(0.11)	0.73(0.12)	0.77	0.71	0.77	0.60
> 30 mmHg (*n* = 88)	0.75(0.11)	0.74(0.06)	0.73(0.13)	0.75(0.11)	0.76	0.69	0.41	0.87
> 40 mmHg (*n* = 63)	0.75(0.08)	0.70(0.08)	0.57(0.20)	0.75(0.08)	0.79	0.69	0.21	0.95

Lung recruitability was defined both as the percent change in not aerated tissue between 5 cmH_2_O and 45 cmH_2_O (recruiters: Δ_45-5_non-aerated tissue > 15%) and as the change in PaO2 between 5 cmH_2_O and 15 cmH_2_O (recruiters: Δ_15-5_PaO2 > 24 mmHg). Performance is evaluated in terms of mean area under the receiver operating characteristic curve (AUC), accuracy (acc), sensitivity (sens) and specificity (spec). Models were trained using the overall dataset (respectively, CT5 + G5 + G15 + M5 + M15 + RPM and CT5 + G5 + M5 + M15 + RPM) and least absolute shrinkage and selection operator (LASSO) algorithm for feature selection

## Discussion

In the present study, the lung recruitability was defined at first as the change in not aerated tissue between 5 cmH_2_O and 45 cmH_2_O to the total lung tissue weight at 5 cmH_2_O, and secondly as Δ_15-5_ PaO_2._ Our findings showed that the best approaches to predict lung recruitment were the one that included lung CT scan taken at 5 cmH_2_O of PEEP. The addition of respiratory mechanics and gas exchange did not significantly improve accuracy.

Concerning ventilatory management in ARDS, lung protective strategies include the application of lung recruitment and adequate PEEP levels to reopen collapsed lung regions by increasing the transpulmonary pressure [37]. The reopening of perfused collapsed/atelectatic lung regions should improve gas exchange, decrease alveolar shunt and ameliorate gas exchange by promoting CO_2_ clearance. However, the increase in transpulmonary pressure, by increasing the end-expiratory lung volume, may also promote lung hyperinflation and higher lung stress at the interface between ventilated and not ventilated lung regions [38].

Among the different monitoring techniques (such as changes in respiratory mechanics, pressure–volume curves, lung ultrasound, electrical impedance tomography), quantitative lung CT analysis, although time-consuming and potentially harmful by exposing the patient to ionizing radiation, remains the most accurate method [6, 7, 11]. By using CT technique, lung recruitment potential is computed as the percentual difference of non-aerated lung tissue at two levels of pressure [8]. Previous studies showed that the application of a machine learning algorithm based on chest radiographs or CT at admission showed a good accuracy in detecting the presence of lung pathologies and ARDS, predicting clinical severity, the need of mechanical ventilation and outcome [15, 16, 24, 39–42]. Zampieri et al., according to a machine learning reanalysis of the ART clinical trial, showed that the application of a recruitment maneuver was associated with higher mortality in ARDS patients with pneumonia compared to sepsis [20]. Thus, the assessment of lung recruitability remains a challenge.

In the present study, we evaluated the possible use of machine learning to predict lung recruitment in ARDS patients, starting from clinical data and CT data obtained at 5 cmH_2_O. The results showed that CT scan at 5 cmH_2_O is the most accurate tool for evaluating lung recruitment and that adding data on lung mechanics and gas exchange does not increase accuracy. When only data on respiratory mechanics were used, low performances were achieved. Thus, while respiratory mechanics monitoring during the course of ARDS remains of paramount importance for VILI prevention and prognostication, our data may suggest that PEEP-induced changes in respiratory mechanics do not yield sufficient information about the potential for lung recruitment and that caution should be adopted when using PEEP-setting strategy based on compliance maximization [4, 43]. When only gas exchange data were used for training, low-to-moderate accuracy was found, suggesting that PEEP-induced changes in PaO_2_ and PaCO_2_ are complex and may suffer from the interference of interdependent physiologic mechanisms [43]. Logistic regression was the preferred and stable machine-learning method for the differentiation of recruiters and non-recruiters when lung recruitability was radiologically defined, whereas random forest was the preferred method when lung recruitability was gas exchange-defined. In this study, parsimonious algorithms with higher potential for clinical use were investigated. According to the frequency with which each feature was selected, ARDS origin was the feature selected with the highest frequency in all the dataset. Respiratory partitioned mechanics features were rarely retained. When considering lung CT features measured at PEEP 5 cmH_2_O, age was always selected to predict radiologically defined lung recruitability. We hypothesize that this may be related to the use of fixed thresholds to define lung aeration compartments, whereas age-related changes of lung volume and tissue density may occur [44].

The best lung recruitment potential cutoff to classify recruiters/non-recruiters remains unknown. In the present study we stratified recruiters and non-recruiters according to the median value of the lung recruitment potential of our whole population (15% and 24 mmHg), as previously suggested [8, 9]. To increase the translatability of the study, we investigated also models classifying recruiters using different thresholds. Comparable results were obtained when using thresholds near the median value of the whole population to define CT-based lung recruitability (10% and 20%, probably because the SMOTE algorithm reduces the impact of classes imbalance); when 30% was used, the worst sensitivity was achieved. On the contrary, using different cutoff to classify recruiters/non-recruiters based on gas exchange data (20 mmHg, 30 mmHg and 40 mmHg), led to worsening specificity and sensitivity, suggesting that the SMOTE algorithm is not sufficient to reduce the impact of classes imbalance and that other techniques should be investigated.

Our study has several strengths. First, it is the first study analyzing a large dataset of CT scans data which showed the possibility to use a machine learning algorithm to divide patients in recruiters and non-recruiters. Secondly, we investigated cross-combinations of three feature selection methods and four classification methods which have commonly been used and achieved high performance in previous studies. Thirdly, if applied these models could avoid the need of a second lung CT scan, decreasing the risk of radiation exposure of the patients and reducing the working time to complete the necessary computation.

However, in this study it was applied an *hybrid* approach with a manual lung segmentation of CT slides, which is time-consuming, with a quantitative analysis and machine learning algorithm. However, automatic lung CT segmentation is now available, which could significantly reduce the radiologic work [45]. The median imputation method was adopted for missing values, as simple and easy interpretable, but more advanced imputation methods [46, 47] can be explored to account for the existing relationship among features. Also, data were collected from a single center and a multicentre study with a large sample size is needed for further validation.

## Conclusions

In conclusion, this study showed the possibility to use a machine learning algorithm based on a single CT imaging at the admission in intensive care unit to classify ARDS patients in responder and not responder to lung recruitment within the first 48 h from the start of mechanical ventilation. The application of this machine learning algorithm with an automatic lung segmentation and quantitative analysis could reduce the computational burden and the ionizing radiation load of the traditional method to assess lung recruitability, helping to improve the tailoring of ventilatory management according to the parenchymal and functional impairment of the ARDS in the acute phase of the disease.

### Supplementary Information

**Additional file 1: Section S1.** Study enrollment process additional details. **Figure S1**. Study protocol flowchart. **Section S2**. Clinical differences in radiologically defined recruiters and non-recruiters in terms of the PEEP test response. **Table S1.** Changes in respiratory mechanics and gas exchange at 5 and 15 cmH_2_O of PEEP in patients divided according to lung potential recruitment (LPR). Continuous data are expressed as mean ± SD or median (interquartile range), while categorical data are expressed as %. Student t test or Mann–Whitney rank-sum tests were used as appropriate. **Section S3.** Additional material and methods. **Figure S2**. Demographic, mechanical, gas exchange and radiological variable considered in during feature selection according to type of variable (M = respiratory system mechanics, G = gas exchange, CT = radiological data, MPR = partitioned respiratory mechanics) and to the measuring condition (5 = 5 cmH_2_O of PEEP, 15 = 15 cmH_2_O of PEEP). **Section S4.** Model performances. **Figure S3.** Validation AUCs for each pair of dataset and ML algorithm. Lung recruitability is defined both from CT (left, recruiters: Δ_45-5_non-aerated tissue > 15%) and from gas exchange data (right, recruiters: Δ_15-5_PaO_2_ > 24 mmHg). The feature selection method applied is marked as _no (no feature selection was applied), corr (feature selection based on correlation was applied), _lasso (the least absolute shrinkage and selection operator was applied once). **Table S2**. Additional metrics on validation and test set of the logistic regression classifier, when lung recruitability was radiologically defined (recruiters: Δ_45-5_non-aerated tissue > 15%). AUC, area under the receiver operating characteristic curve; acc, accuracy; sens, sensitivity; spec, specificity. M5: lung mechanics at PEEP 5 cmH_2_O; M15: lung mechanics at PEEP 15 cmH_2_O; RPM: respiratory partitioned mechanics; G5: gas exchange measured at PEEP 5 cmH_2_O; G15: gas exchange measured at PEEP 15 cmH_2_O; CT5: CT imaging acquired at PEEP 5 cmH_2_O. **Table S3**. Additional metrics on validation and test set, reported for the random forest classifier, when lung recruitability was gas exchange-defined (recruiters: Δ_15-5_PaO_2_ > 24 mmHg).

## Data Availability

The dataset is available from the corresponding author on reasonable request.

## References

[1] Gattinoni L, Marini JJ, Pesenti A, Quintel M, Mancebo J, Brochard L (2016). The, “baby lung” became an adult. Intensive Care Med.

[2] Fan E, Del Sorbo L, Goligher EC, Hodgson CL, Munshi L, Walkey AJ (2017). An official American Thoracic Society/European Society of intensive care medicine/society of critical care medicine clinical practice guideline: mechanical ventilation in adult patients with acute respiratory distress syndrome. Am J Respir Crit Care Med.

[3] Pensier J, de Jong A, Hajjej Z, Molinari N, Carr J, Belafia F (2019). Effect of lung recruitment maneuver on oxygenation, physiological parameters and mortality in acute respiratory distress syndrome patients: a systematic review and meta-analysis. Intensive Care Med.

[4] Cavalcanti AB, Suzumura ÉA, Laranjeira LN, De Moraes PD, Damiani LP, Guimarães HP (2017). Effect of lung recruitment and titrated Positive End-Expiratory Pressure (PEEP) vs low PEEP on mortality in patients with acute respiratory distress syndrome—a randomized clinical trial. JAMA.

[5] Dianti J, Tisminetzky M, Ferreyro BL, Englesakis M, Del Sorbo L, Sud S (2022). Association of positive end-expiratory pressure and lung recruitment selection strategies with mortality in acute respiratory distress syndrome: a systematic review and network meta-analysis. Am J Respir Crit Care Med.

[6] Chiumello D, Papa GFS, Artigas A, Bouhemad B, Grgic A, Heunks L (2019). ERS statement on chest imaging in acute respiratory failure. Eur Respir J.

[7] Gattinoni L, Caironi P, Pelosi P, Goodman LR (2001). What has computed tomography taught us about the acute respiratory distress syndrome?. Am J Respir Crit Care Med.

[8] Gattinoni L, Caironi P, Cressoni M, Chiumello D, Ranieri VM, Quintel M (2006). Lung recruitment in patients with the acute respiratory distress syndrome. N Engl J Med.

[9] Coppola S, Froio S, Marino A, Brioni M, Cesana BM, Cressoni M (2019). Respiratory mechanics, lung recruitability, and gas exchange in pulmonary and extrapulmonary acute respiratory distress syndrome. Crit Care Med.

[10] Pierrakos C, Smit MR, Hagens LA, Heijnen NFL, Hollmann MW, Schultz MJ (2021). Assessment of the effect of recruitment maneuver on lung aeration through imaging analysis in invasively ventilated patients: a systematic review. Front Physiol.

[11] Chiumello D, Marino A, Brioni M, Cigada I, Menga F, Colombo A (2016). Lung recruitment assessed by respiratory mechanics and computed tomography in patients with acute respiratory distress syndrome what is the relationship?. Am J Respir Crit Care Med.

[12] Cressoni M, Chiumello D, Carlesso E, Chiurazzi C, Amini M, Brioni M (2014). Compressive forces and computed tomography-derived positive end-expiratory pressure in acute respiratory distress syndrome. Anesthesiology.

[13] Mlodzinski E, Stone DJ, Celi LA (2020). Machine learning for pulmonary and critical care medicine: a narrative review. Pulm Ther.

[14] Wong AKI, Cheung PC, Kamaleswaran R, Martin GS, Holder AL (2020). Machine learning methods to predict acute respiratory failure and acute respiratory distress syndrome. Front Big Data.

[15] Maddali MV, Churpek M, Pham T, Rezoagli E, Zhuo H, Zhao W (2022). Validation and utility of ARDS subphenotypes identified by machine-learning models using clinical data: an observational, multicohort, retrospective analysis. Lancet Respir Med.

[16] Sinha P, Churpek MM, Calfee CS (2020). Machine learning classifier models can identify acute respiratory distress syndrome phenotypes using readily available clinical data. Am J Respir Crit Care Med.

[17] Sayed M, Riaño D, Villar J (2021). Novel criteria to classify ARDS severity using a machine learning approach. Crit Care.

[18] Parreco J, Hidalgo A, Parks JJ, Kozol R, Rattan R (2018). Using artificial intelligence to predict prolonged mechanical ventilation and tracheostomy placement. J Surg Res.

[19] Mamandipoor B, Frutos-Vivar F, Peñuelas O, Rezar R, Raymondos K, Muriel A (2021). Machine learning predicts mortality based on analysis of ventilation parameters of critically ill patients: multi-centre validation. BMC Med Inform Decis Mak.

[20] Zampieri FG, Costa EL, Iwashyna TJ, Carvalho CRR, Damiani LP, Taniguchi LU (2019). Heterogeneous effects of alveolar recruitment in acute respiratory distress syndrome: a machine learning reanalysis of the alveolar recruitment for acute respiratory distress syndrome trial. Br J Anaesth.

[21] Wang S, Zha Y, Li W, Wu Q, Li X, Niu M (2020). A fully automatic deep learning system for COVID-19 diagnostic and prognostic analysis. Eur Respir J.

[22] Wu C, Chen X, Cai Y, Xia J, Zhou X, Xu S (2020). Risk factors associated with acute respiratory distress syndrome and death in patients with coronavirus disease 2019 pneumonia in Wuhan. China JAMA Intern Med.

[23] Saba L, Agarwal M, Patrick A, Puvvula A, Gupta SK, Carriero A (2021). Six artificial intelligence paradigms for tissue characterisation and classification of non-COVID-19 pneumonia against COVID-19 pneumonia in computed tomography lungs. Int J Comput Assist Radiol Surg.

[24] Chieregato M, Frangiamore F, Morassi M, Baresi C, Nici S, Bassetti C (2022). A hybrid machine learning/deep learning COVID-19 severity predictive model from CT images and clinical data. Sci Rep.

[25] Wu Q, Wang S, Li L, Wu Q, Qian W, Hu Y (2020). Radiomics analysis of computed tomography helps predict poor prognostic outcome in COVID-19. Theranostics.

[26] Coppola S, Caccioppola A, Froio S, Formenti P, De Giorgis V, Galanti V (2020). Effect of mechanical power on intensive care mortality in ARDS patients. Crit Care.

[27] Chiumello D, Consonni D, Coppola S, Froio S, Crimella F, Colombo A (2016). The occlusion tests and end-expiratory esophageal pressure: measurements and comparison in controlled and assisted ventilation. Ann Intensive Care.

[28] Scikit-learn: Machine Learning in Python, Pedregosa et al., JMLR 12, 2011:2825-2830.

[29] McKinney W, others. Data structures for statistical computing in python. In: Proceedings of the 9th Python in Science Conference. 2010:51–6.

[30] Chawla NV, Bowyer KW, Hall LO, Kegelmeyer WP (2002). SMOTE: synthetic minority over-sampling technique. J Artif Intell Res.

[31] Cortes C, Vapnik V (1995). Support-vector networks. Mach Learn.

[32] Breiman L (2001). Random forest. Mach Learn.

[33] Chen T, Guestrin C. XGBoost. In: Proceedings of the 22nd ACM SIGKDD International Conference on Knowledge Discovery and Data Mining. New York, NY, USA: ACM; 2016. p. 785–94.

[34] Zhou ZH (2012). Ensemble methods. Foundation and algorithms.

[35] Sagi O, Rokach L (2018). Ensemble learning: A survey. WIREs Data Min Knowl Discov.

[36] Hsu CW, Chang CC, Lin CJ. A Practical Guide to Support Vector Classification. Engineering. 2016;321–32.

[37] Lachmann B (1992). Open up the lung and keep the lung open. Intensive Care Med.

[38] Chiumello D, Carlesso E, Cadringher P, Caironi P, Valenza F, Polli F (2008). Lung stress and strain during mechanical ventilation for acute respiratory distress syndrome. Am J Respir Crit Care Med.

[39] Sjoding MW, Taylor D, Motyka J, Lee E, Co I, Claar D (2021). Deep learning to detect acute respiratory distress syndrome on chest radiographs: a retrospective study with external validation. Lancet Digit Health.

[40] Röhrich S, Hofmanninger J, Negrin L, Langs G, Prosch H (2021). Radiomics score predicts acute respiratory distress syndrome based on the initial CT scan after trauma. Eur Radiol.

[41] Reamaroon N, Sjoding MW, Lin K, Iwashyna TJ, Najarian K (2019). Accounting for label uncertainty in machine learning for detection of acute respiratory distress syndrome. IEEE J Biomed Health Inform.

[42] Reamaroon N, Sjoding MW, Gryak J, Athey BD, Najarian K, Derksen H (2021). Automated detection of acute respiratory distress syndrome from chest X-rays using directionality Measure and deep learning features. Comput Biol Med.

[43] Grieco DL, De Pascale GAM (2020). Lung recruitability and positive end-expiratory pressure setting in ARDS caused by COVID-19. Ann Oncol.

[44] Dicente Cid Y, Mamonov A, Beers A, Thomas A, Kovalev V, Kalpathy-Cramer J, et al. Making sense of large data sets without annotations: analyzing age-related correlations from lung CT scans. Medical Imaging 2017: Imaging Informatics for Healthcare, Research, and Applications. 2017; 10138: 52–63

[45] Klapsing P, Herrmann P, Quintel M, Moerer O (2017). Automatic quantitative computed tomography segmentation and analysis of aerated lung volumes in acute respiratory distress syndrome—a comparative diagnostic study. J Crit Care.

[46] Beretta L, Santaniello A (2016). Nearest neighbor imputation algorithms: a critical evaluation. BMC Med Inform Decis Mak.

[47] Kim JC, Chung K (2020). Multi-modal stacked denoising autoencoder for handling missing data in healthcare big data. IEEE Access.

