# Supplementary material for: Machine learning predicts lung recruitment in acute respiratory distress syndrome using single lung CT scan
Source: Ann Intensive Care. 2023 Jul 5;13:60. doi: 10.1186/s13613-023-01154-5 (PMC10322807; doi:10.1186/s13613-023-01154-5)
Supplement: Supplementary file 1 — Additional file 1: Section S1. Study enrollment process additional details. Figure S1. Study protocol flowchart. Section S2. Clinical differences in radiologically defined recruiters and non-recruiters in terms of the PEEP test response. Table S1. Changes in respiratory mechanics and gas exchange at 5 and 15 cmH2O of PEEP in patients divided according to lung potential recruitment (LPR). Continuous data are expressed as mean ± SD or median (interquartile range), while categorical data are expressed as %. Student t test or Mann–Whitney rank-sum tests were used as appropriate. Section S3. Additional material and methods. Figure S2. Demographic, mechanical, gas exchange and radiological variable considered in during feature selection according to type of variable (M = respiratory system mechanics, G = gas exchange, CT = radiological data, MPR = partitioned respiratory mechanics) and to the measuring condition (5 = 5 cmH2O of PEEP, 15 = 15 cmH2O of PEEP). Section S4. Model performances. Figure S3. Validation AUCs for each pair of dataset and ML algorithm. Lung recruitability is defined both from CT (left, recruiters: Δ45-5non-aerated tissue > 15%) and from gas exchange data (right, recruiters: Δ15-5PaO2 > 24 mmHg). The feature selection method applied is marked as _no (no feature selection was applied), corr (feature selection based on correlation was applied), _lasso (the least absolute shrinkage and selection operator was applied once). Table S2. Additional metrics on validation and test set of the logistic regression classifier, when lung recruitability was radiologically defined (recruiters: Δ45-5non-aerated tissue > 15%). AUC, area under the receiver operating characteristic curve; acc, accuracy; sens, sensitivity; spec, specificity. M5: lung mechanics at PEEP 5 cmH2O; M15: lung mechanics at PEEP 15 cmH2O; RPM: respiratory partitioned mechanics; G5: gas exchange measured at PEEP 5 cmH2O; G15: gas exchange measured at PEEP 15 cmH2O; CT5: CT imaging acquired at PEEP [file 13613_2023_1154_MOESM1_ESM.docx]

**MACHINE LEARNING PREDICTS LUNG RECRUITMENT IN ACUTE RESPIRATORY DISTRESS SYNDROME USING SINGLE LUNG CT SCAN**

Francesca Pennati MS^1^, Andrea Aliverti MS^1^, Tommaso Pozzi MD^2^, Simone Gattarello MD^3^, Fabio Lombardo MD^3^, Silvia Coppola MD^4^ and Davide Chiumello MD^2,4,5^

^1^Dipartimento di Elettronica, Informazione e Bioingegneria, Politecnico di Milano, Milan, Italy

Department of Anesthesia and Intensive Care, ASST Santi Paolo e Carlo, San Paolo University Hospital, Via Di Rudini 9 Milan, Italy

Department of Health Sciences, University of Milan, Milan, Italy

Coordinated Research Center on Respiratory Failure, University of Milan, Milan, Italy

**Corresponding Author**

Prof. Davide Chiumello

Department of Anesthesia and Intensive Care, ASST Santi Paolo e Carlo, San Paolo University Hospital, Via Di Rudini 9 Milan, Italy

E-mail address: [davide.chiumello@unimi.it](mailto:davide.chiumello@unimi.it)

**Section 1.** Study enrolment process additional details.

**Figure S1**. Study protocol flow chart.


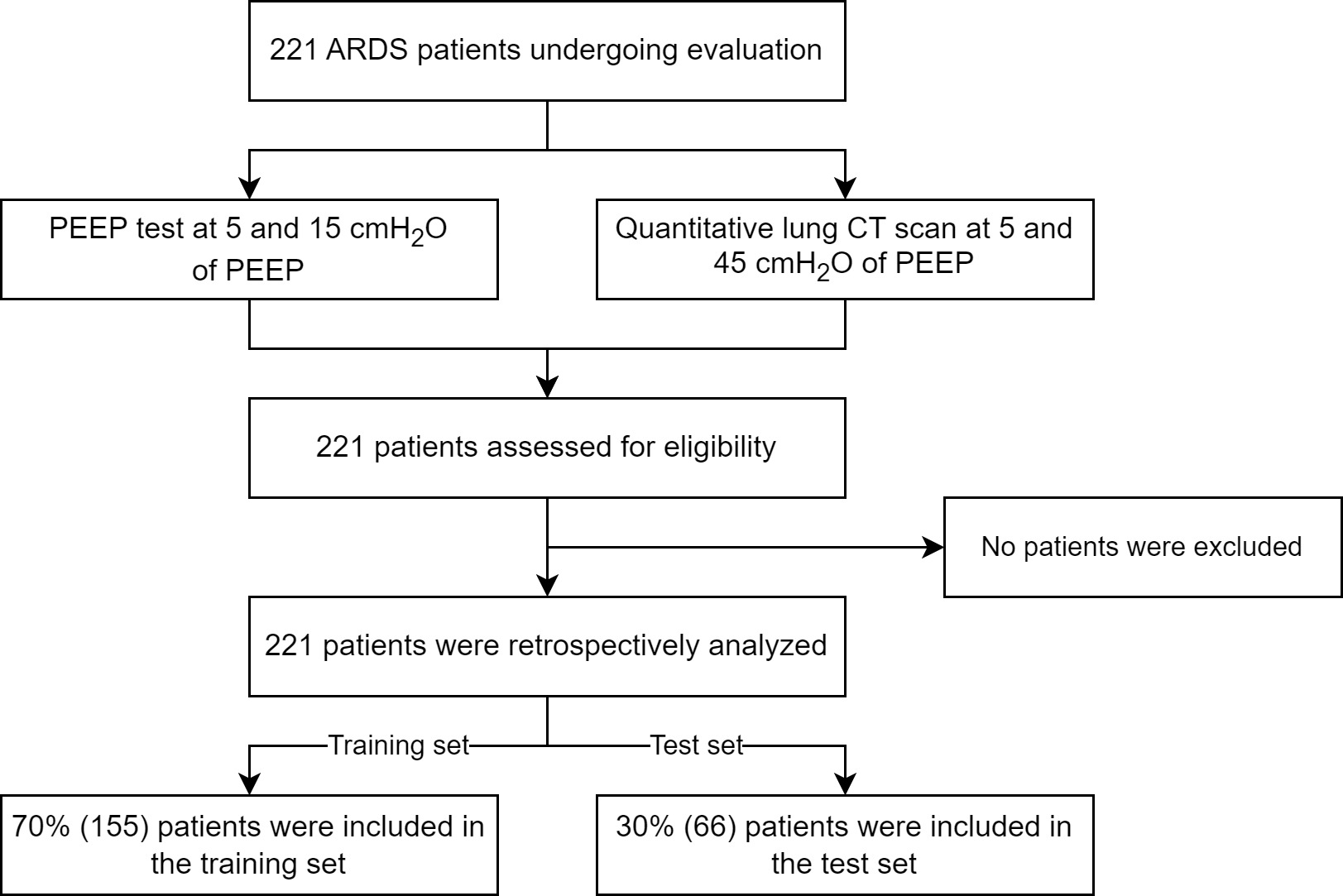


**Section 2**. Clinical differences in radiologically-defined recruiters and non recruiters in terms of the PEEP test response.

PEEP test (Table S1)

From PEEP 5 to 15 cmH_2_O the improvement in oxygenation (Δ_15-5_PaO_2_/FiO_2_) was significantly higher in recruiters (57 [28 – 101] *vs* 37 [8 – 79] mmHg).

**Table S1.** Changes in respiratory mechanics and gas exchange at 5 and 15 cmH_2_O of PEEP in patients divided according to lung potential recruitment (LPR). Continuous data are expressed as mean ± SD or median (interquartile range), while categorical data are expressed as %. Student t test or Mann–Whitney rank-sum tests were used as appropriate.

| **Variables** | **Recruiters (LPR>15%)** | **Non-recruiters (LPR≤15%)** | **p-value** |
| --- | --- | --- | --- |
| **Respiratory mechanics** | | | |
| Δ_15-5_ Driving pressure (cmH_2_O) | 0.2 (-1.4, 2.3) | 0.0 (-2.0, 1.1) | 0.092 |
| Δ_15-5_ Respiratory system elastance (cmH_2_O/L) | 0.1 (-2.8, 4.5) | -0.6 (-4.0, 2.0) | 0.101 |
| Δ_15-5_ Mechanical Power (J/min) | -6.7 (-8.4, -5.0) | -7.1 (-9.7, -5.6) | 0.050 |
| **Respiratory partitioned mechanics** | | | |
| Δ_15-5_ Lung elastance (cmH_2_O/L) | -0.4 (-3.3, 4.5) | -0.2 (-3.1, 3.1) | 0.905 |
| Δ_15-5_ Chest wall elastance (cmH_2_O/L) | -0.5 (-2.1, 0.9) | -0.7 (-2.9, 1.9) | 0.713 |
| **Gas exchange** | | | |
| Δ_15-5_ PaCO_2_ (mmHg) | 0.1 (-1.9, 2.2) | 0.6 (-1.2, 2.7) | 0.253 |
| Δ_15-5_ PaO_2_ (mmHg) | 32 (17, 58) | 18 (3, 32) | **<0.001** |
| Δ_15-5_ PaO_2_/FiO_2_ | 57 (28, 101) | 37 (8, 79) | **<0.001** |

**Section 3.** Additional material and methods.

**Figure S2**. Demographic, mechanical, gas exchange and radiological variable considered in during feature selection according to type of variable (M = respiratory system mechanics, G = gas exchange, CT = radiological data, MPR = partitioned respiratory mechanics) and to the measuring condition (5 = 5 cmH_2_O of PEEP, 15 = 15 cmH_2_O of PEEP).


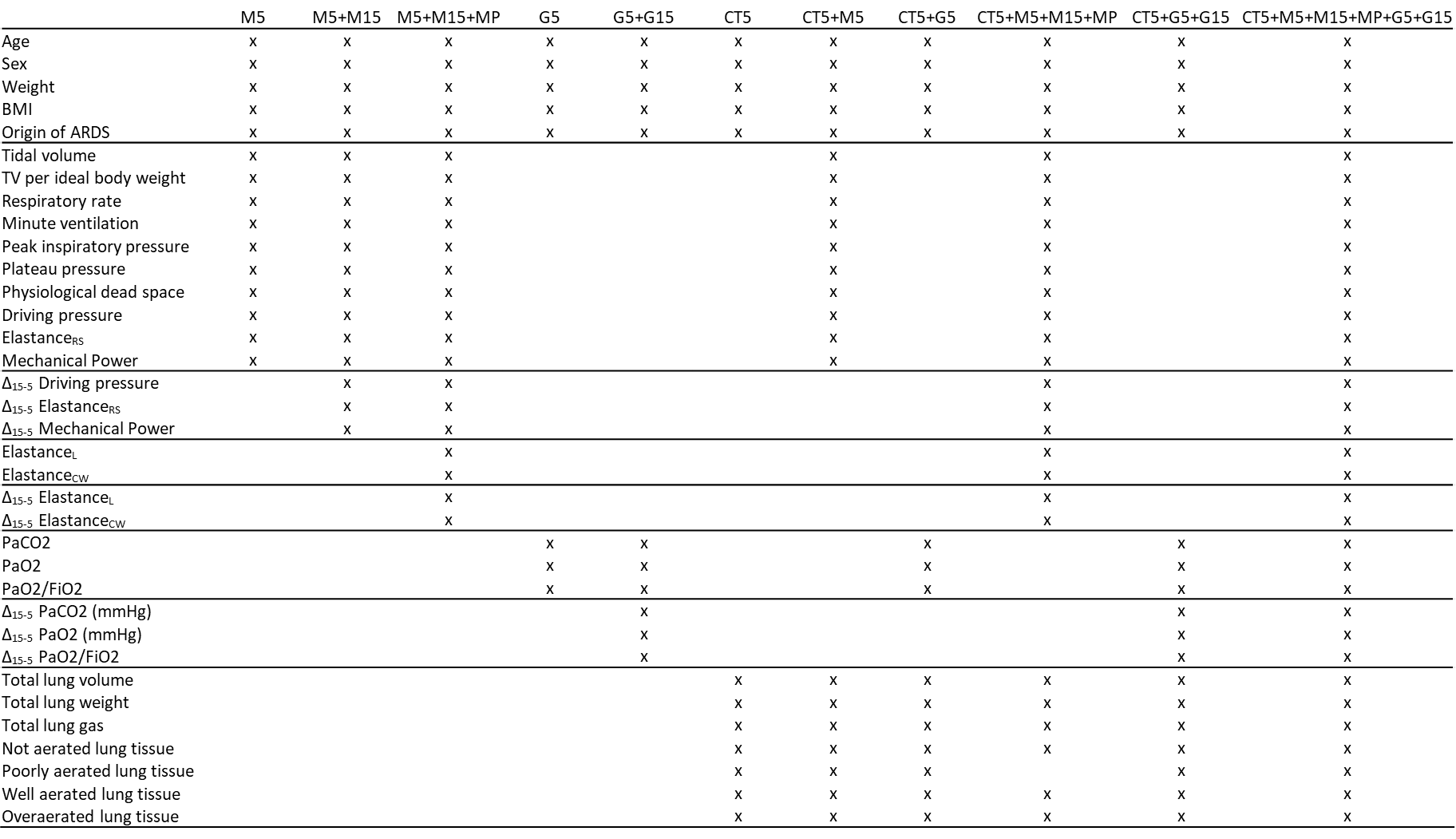


**LASSO algorithm details.** In the training set, the least absolute shrinkage and selection operator (LASSO) was repeatedly applied. A grid search strategy with a stratified five-fold cross validation repeated 10 times was performed to optimize the regularization parameter λ. The maximum number of features accepted in a model was set to be lower than 10% of the sample size (1). The features selected were then used to train the final model on the entire training set. To generate parsimonious algorithms with higher potential for clinical use, sequential models were generated with increased values of the tuning parameter λ and selecting the smallest value of λ that gave a model with fewer than 5 variables. After repeating this procedure ten times, each time with a different random data split, features were retained according to the frequency with which they were chosen, maintaining the features that had been selected in more than 50% of the cases. For comparison an unsupervised method based on correlation was evaluated. The results are shown in figure S2.

**Development of classification models with LASSO-processed sets of variables**. When lung recruitability was defined according to radiologically scan as the percent change in not aerated tissue between 5 cmH_2_O and 45 cmH_2_O, ARDS origin (pulmonary/extra-pulmonary) was the feature retained in all the dataset. Tidal volume, airway plateau pressure and driving pressure were the most frequent selected features when the model included only respiratory system mechanics measured at PEEP 5 cmH_2_O, whereas if lung mechanics at PEEP 15 cmH_2_O was included (dataset M5+M15), the Δ_15-5_ Mechanical Power was also retained. If respiratory partitioned mechanics was included (dataset M5+M15+RPM), also lung elastance was retained. When the model included only gas exchange parameters (G5), PaO_2_/FiO_2_ was the most frequent selected feature, whereas if gas exchange at PEEP 15 cmH_2_O was considered (G5+G15), Δ_15-5_PaO_2_ was also retained. Age, well and not aerated tissue were always selected when the model included only CT parameters (CT5). PaO_2_/FiO_2_, Δ_15-5_ Mechanical Power and Δ_15-5_ PaO_2_ were the features retained when mechanical and gas exchange features were added to CT5 (CT5+G5+G15).

When lung recruitability was defined based on gas exchange (Δ_15-5_PaO_2_)_,_ ARDS origin was again retained in all the models. If respiratory system mechanics parameters measured at PEEP 5 cmH_2_O (M5) were included, BMI and mechanical power were the most frequently selected features. When lung mechanics at PEEP 15 cmH_2_O (M5+M15) and respiratory partitioned mechanics (M5+M15+RPM) were added, Δ_15-5_ Driving Pressure and chest wall elastance were respectively retained. When the model included only gas exchange parameters (G5), BMI and PaO_2_ were the most frequently selected feature. When the model included only CT parameters (CT5), total, well and poorly aerated tissue were always selected. PaO_2_, Δ_15-5_ Driving Pressure and chest wall elastance were retained when mechanical and gas exchange features were added to CT5 (CT5+G5+M5+M15+RPM).

**Section 4.** Model performances.

**Figure S3.** Validation AUCs for each pair of dataset and ML algorithm. Lung recruitability is defined both from CT (left, recruiters: Δ_45-5_non-aerated tissue > 15%) and from gas exchange data (right, recruiters: Δ_15-5_PaO_2_ > 24mmHg). The feature selection method applied is marked as _no (no feature selection was applied), corr (feature selection based on correlation was applied), _lasso (the least absolute shrinkage and selection operator was applied once).


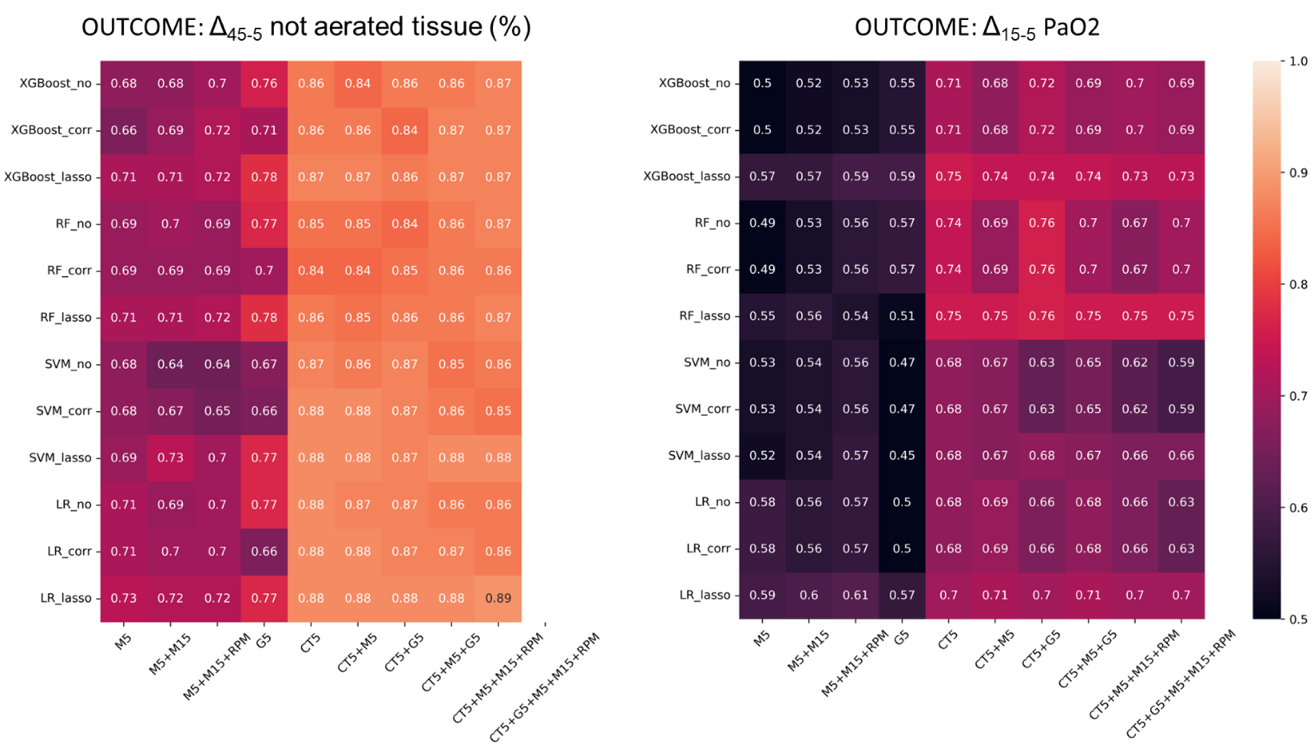


**Section 4.** Model performances

*Radiologically-defined lung recruitability: ML algorithm selection*

When lung recruitability was defined from CT, the Cochran test reported a statistically significant difference between the classifiers on mechanical models (M5 _LASSO_, p=0.02; M5 _LASSO_ +M15 _LASSO_, p<0.001; M5 _LASSO_ +M15 _LASSO_ +RPM _LASSO_, p<0.001), with McNemars test indicating significant differences between logistic regression and random forest and between logistic regression and gradient-boosted tree. No statistically significant difference was reported between classifiers based on gas exchange parameters. On CT models, logistic regression reported the highest validation AUCs. However, statistically significant differences were found only on two models including CT data (CT5 _LASSO_ +G5 _LASSO_, p=0.023 and GT5 _LASSO_ +G5 _LASSO_ +M5 _LASSO_, p=0.041), with McNemars test indicating significant differences between logistic regression and gradient-boosted tree in both the models.

*Gas exchange-defined lung recruitability: ML algorithm selection*

When lung recruitability was defined based on gas exchange (Recruiting patients: Δ_15-5_PaO_2_ > 24mmHg) (Figure 3), random forest resulted in the highest validation AUCs on gas exchange and CT models. The Cochran test reported statistically significant difference between the classifiers based on gas exchange parameters (p<0.001) and CT parameters (p<0.001), with McNemars test indicating significant differences between random forest and logistic regression and between random forest and support vector machine in all the models.

**Table S2**. Additional metrics on validation and test set of the logistic regression classifier, when lung recruitability was radiologically-defined (recruiters: Δ_45-5_non-aerated tissue > 15%). AUC, area under the receiver operating characteristic curve; acc, accuracy; sens, sensitivity; spec, specificity. M5: lung mechanics at PEEP 5 cmH_2_O; M15: lung mechanics at PEEP 15 cmH_2_O; RPM: respiratory partitioned mechanics; G5: gas exchange measured at PEEP 5 cmH_2_O; G15: gas exchange measured at PEEP 15 cmH_2_O; CT5: CT imaging acquired at PEEP 5 cmH_2_O.

|  | VALIDATION | | | | TEST | | | |
| --- | --- | --- | --- | --- | --- | --- | --- | --- |
|  | AUC | acc | Sens | Spec | AUC | acc | Sens | Spec |
| M5 | 0.72(0.09) | 0.66(0.08) | 0.70(0.11) | 0.63(0.10) | 0.67 | 0.66 | 0.71 | 0.58 |
| M5+M15 | 0.72(0.08) | 0.65(0.07) | 0.67(0.12) | 0.64(0.09) | 0.66 | 0.63 | 0.66 | 0.58 |
| M5+M15+RPM | 0.73(0.10) | 0.67(0.09) | 0.69(0.13) | 0.65(0.11) | 0.65 | 0.63 | 0.66 | 0.58 |
| G5 | 0.77(0.07) | 0.66(0.08) | 0.71(0.12) | 0.62(0.12) | 0.69 | 0.66 | 0.78 | 0.46 |
| G5+G15 | 0.78(0.07) | 0.68(0.07) | 0.71(0.11) | 0.64(0.11) | 0.75 | 0.72 | 0.78 | 0.62 |
| CT5 | 0.89(0.04) | 0.81(0.06) | 0.79(0.09) | 0.82(0.09) | 0.88 | 0.84 | 0.88 | 0.77 |
| CT5+M5 | 0.88(0.07) | 0.81(0.07) | 0.80(0.12) | 0.82(0.07) | 0.88 | 0.84 | 0.90 | 0.73 |
| CT5+G5 | 0.88(0.06) | 0.81(0.05) | 0.79(0.10) | 0.82(0.07) | 0.88 | 0.84 | 0.88 | 0.77 |
| CT5+M5+G5 | 0.88(0.06) | 0.81(0.07) | 0.79(0.11) | 0.82(0.08) | 0.88 | 0.84 | 0.90 | 0.81 |
| CT5+G5+G15 | 0.89(0.05) | 0.81(0.06) | 0.79(0.11) | 0.83(0.09) | 0.89 | 0.87 | 0.90 | 0.81 |
| CT5+M5+M15+MP | 0.89(0.05) | 0.81(0.06) | 0.79(0.10) | 0.82(0.07) | 0.87 | 0.81 | 0.83 | 0.77 |
| CT5+M5+M15+RPM G5+G15 | 0.90(0.05) | 0.80(0.05) | 0.79(0.11) | 0.82(0.10) | 0.89 | 0.82 | 0.85 | 0.77 |

**Table S3**. Additional metrics on validation and test set, reported for the random forest classifier, when lung recruitability was gas exchange-defined (recruiters: Δ_15-5_PaO_2_ > 24mmHg).

AUC, area under the receiver operating characteristic curve; acc, accuracy; sens, sensitivity; spec, specificity. M5: lung mechanics at PEEP 5 cmH_2_O; M15: lung mechanics at PEEP 15 cmH_2_O; RPM: respiratory partitioned mechanics; G5: gas exchange measured at PEEP 5 cmH_2_O; G15: gas exchange measured at PEEP 15 cmH_2_O; CT5: CT imaging acquired at PEEP 5 cmH_2_O.

|  | VALIDATION | | | | TEST | | | |
| --- | --- | --- | --- | --- | --- | --- | --- | --- |
|  | AUC | acc | Sens | Spec | AUC | acc | Sens | Spec |
| M5 | 0.55(0.10) | 0.53(0.08) | 0.46(0.12) | 0.59(0.17) | 0.49 | 0.51 | 0.22 | 0.84 |
| M5+M15 | 0.56(0.09) | 0.56(0.08) | 0.58(0.17) | 0.60(0.13) | 0.59 | 0.52 | 0.25 | 0.84 |
| M5+M15+RPM | 0.57(0.10) | 0.56(0.09) | 0.52(0.12) | 0.60(0.13) | 0.60 | 0.48 | 0.25 | 0.74 |
| G5 | 0.59(0.08) | 0.56(0.08) | 0.48(0.15) | 0.62(0.14) | 0.50 | 0.43 | 0.28 | 0.61 |
| CT5 | 0.77(0.07) | 0.71(0.08) | 0.73(0.12) | 0.70(0.15) | 0.81 | 0.79 | 0.75 | 0.84 |
| CT5+M5 | 0.76(0.06) | 0.69 (0.07) | 0.71(0.12) | 0.68(0.13) | 0.81 | 0.76 | 0.75 | 0.77 |
| CT5+G5 | 0.78(0.07) | 0.71(0.07) | 0.73(0.12) | 0.70(0.13) | 0.80 | 0.76 | 0.72 | 0.81 |
| CT5+M5+G5 | 0.78(0.07) | 0.71(0.07) | 0.74(0.13) | 0.68(0.12) | 0.80 | 0.79 | 0.78 | 0.81 |
| CT5+M5+M15+RPM | 0.76(0.06) | 0.69(0.07) | 0.72(0.12) | 0.66(0.13) | 0.81 | 0.76 | 0.72 | 0.81 |
| CT5+M5+M15+RPM+G5 | 0.78(0.07) | 0.70(0.08) | 0.73(0.12) | 0.68(0.12) | 0.79 | 0.73 | 0.67 | 0.81 |

References

1. Jain AK, Duin PW, Jianchang Mao. Statistical pattern recognition: a review. IEEE Trans Pattern Anal Mach Intell. 2000;22(1):4–37.
